# The gene knockout of angiotensin II type 1a receptor improves high-fat diet-induced obesity in rat *via* promoting adipose lipolysis

**DOI:** 10.1371/journal.pone.0267331

**Published:** 2022-07-08

**Authors:** Aiyun Li, Wenjuan Shi, Jin Wang, Xuejiao Wang, Yan Zhang, Zhandong Lei, Xiang-Ying Jiao

**Affiliations:** Key Laboratory of Cellular Physiology (Shanxi Medical University), Ministry of Education, and The Department of Physiology, Shanxi Medical University, Taiyuan, China; Tohoku University, JAPAN

## Abstract

**Aims:**

The renin-angiotensin system (RAS) is over-activated and the serum angiotensin II (Ang II) level increased in obese patients, while their correlations were incompletely understood. This study aims to explore the role of Ang II in diet-induced obesity by focusing on adipose lipid anabolism and catabolism.

**Methods:**

Rat model of AT1aR gene knockout were established to investigate the special role of Ang II on adipose lipid metabolism. Wild-type (WT) and AT1aR gene knockout (AT1aR^-/-^) SD rats were fed with normal diet or high-fat diet for 12 weeks. Adipose morphology and adipose lipid synthesis and lipolysis were examined.

**Results:**

AT1aR deficiency activated lipolysis-related enzymes and increased the levels of NEFAs and glycerol released from adipose tissue in high-fat diet rats, while did not affect triglycerides synthesis. Besides, AT1aR knockout promoted energy expenditure and fatty acids oxidation in adipose tissue. cAMP levels and PKA phosphorylation in the adipose tissue were significantly increased in AT1aR^-/-^ rats fed with high-fat. Activated PKA could promote adipose lipolysis and thus improved adipose histomorphology and insulin sensitivity in high-fat diet rats.

**Conclusions:**

AT1aR deficiency alleviated adipocyte hypertrophy in high-fat diet rats by promoting adipose lipolysis probably via cAMP/PKA pathway, and thereby delayed the onset of obesity and related metabolic diseases.

## 1. Introduction

Obesity is a growing health problem that induces major metabolic disorders, such as diabetes, cardiovascular disease, and hypertension. Obesity has been officially listed as a disease by the World Health Organization in 2000. It has been predicted that more than 57.8% adults and about 3.3 billion people worldwide will facing overweight or obesity problems by 2030 [1]. With an increasing prevalence of obesity, it is urgent to reveal the pathophysiological mechanism for obesity and develop effective therapy strategies for treatment of obesity and its associated disorders.

Adipose tissue plays major role in the development of obesity. Excess energy is stored in the adipose tissue in the form of triacylglycerol (TAG), while fatty acid mobilization via lipolysis releases fatty acids for energy supply [2]. Physiologically, adipose tissue maintains a dynamic balance between lipid synthesis and decomposition. However, adipose tissue stores excess energy by expansion and remodeling when the storage capacity of adipocytes is exceeded in response to overfeeding, resulting in fat accumulation and adipocyte hypertrophy [3, 4]. Therefore, inhibiting adipocyte hypertrophy can be an effective strategy for obesity treatment.

The renin-angiotensin system (RAS) is a dynamic physiologic system and its classic function is to regulate blood pressure and fluid and electrolyte balance. Angiotensin-converting enzyme inhibitor (ACEI) and angiotensin II receptor blockers (ARB) has been clinically used as antihypertensive drugs and play a crucial role in the treatment of cardiovascular diseases such as hypertension and heart failure. However, it has been found that these drugs have a positive effect in metabolic diseases such as obesity, insulin resistance and diabetes [5, 6], indicating the relations between RAS and metabolic diseases. Animal studies have shown that mice with renin gene knockout are resistant to diet-induced obesity [7]. Besides, excessive activation of RAS is a common feature in obese patients [8, 9], and the serum levels of angiotensinogen (AGT) and angiotensin II (Ang II) in the obese are higher than those in normal population [10–12]. Adipose tissue is the most abundant source of AGT outside liver. It is showed that AGT expression increased in the adipose tissue of obese animal models and adipocyte-specific enhancement of AGT lead to insulin resistance [13], indicating the special role of adipose tissue RAS in regulation of metabolic homeostasis. Adipose RAS has attracted more and more attention due to its close relationship with obesity and adipose dysfunction over the years [14–16]. Previously Ryuji Kouyama et al. have showed that systemic AT1aR deficiency attenuated high fat diet-induced weight gain and adiposity in mice [17]. However, the underline mechanism by which AT1aR deficiency modulates high fat-diet induced obesity and insulin resistance remain unclear, especially in the context of adipose tissue.

As the predominant peptide of RAS, angiotensin II (Ang II) exerts effects mainly by binding with angiotensin type 1 receptor (AT1R) in adipose tissue [14]. In the current study, we established an obese rat model with AT1aR gene knockout to explore the role of adipose RAS in the develop process of obesity. The results showed that the gene knockout of AT1aR ameliorated adipocyte hypertrophy by promoting adipose lipolysis through cAMP/PKA pathway, and thereby improving obesity and related metabolic disorders. Our finding suggests that targeting RAS signaling in adipose tissue may become a promising therapy avenue for obesity and associated metabolic abnormalities.

## 2. Materials and methods

### 2.1 Experimental animals

Wild type male Sprague-Dawley (SD) rats purchased from the Experimental Animal Center of Shanxi Medical University and AT1aR^-/-^ male SD rats (Nanjing University-Nanjing Institute of Biology) were all fed in the SPF laboratory animal environmental facilities with 12 h light/dark cycles under standard room temperature (22 ± 2°C) and free access to water and food. The sgRNA & CRISPR/Cas9 system (Table 1) were used to generated AT1aR systemic deficiency rats and the homozygous rats have been verified by polymerase chain reaction (PCR) and further confirmed by the detection of AT1aR expression in major Ang II responsive tissues using RT-PCR method (S1 Fig). All animal experiments were in accordance with the guidelines for the management of animals for medical experiments issued by the Ministry of Health of the People’s Republic of China (No. 55) and animal ethics standards of Shanxi Medical University, and approved by the ethics committee.

**Table 1:** sgRNA primer sequences for AT1aR^-/-^ rats generation.

sgRNA Name	sgRNA Primer (5’-3’)	PAM
Agtr1a-S7	GGTCTGAAGCATAGCTCGGT	TGG
Agtr1a-S8	GTGACTCAGTTACTGGTCCT	TGG
Agtr1a-S9	ATGGGCCACATACCTACGTG	TGG
Agtr1a-S10	TCTCCACTGCTAATGACTAC	AGG

4-week male wild type (WT) rats and AT1aR^-/-^ rats were randomly divided into normal diet group (ND) and high-fat-diet group (HFD). The HFD group rats were fed with 60% high-fat feed (D12492, Whitby Technology Co., Ltd. Beijing, China) and the ND group rats were fed with normal feed for 12 weeks. Food intake and body weight was recorded weekly. At the end of feeding, rats were fasted overnight and anesthetized with an intraperitoneal injection of pentobarbital, blood sample was collected from the abdominal aorta for the assay of blood glucose, nonesterified free fatty acids (NEFAs), triglyceride (TG), total cholesterol (T-CHO), low-density lipoprotein (LDL) and high-density lipoprotein (HDL) contents with commercial kits (Jiancheng Bioengineering Institute, Nanjing, China). Serum glycerol content was measured with glycerol (liquid sample) enzymatic determination kit (Applygen Gene Technology Co., Ltd, Beijing). Epididymal adipose tissue were isolated immediately or stored at minus 80 degrees Celsius until analysis. Epididymal fat index were calculated as the ratio of epididymal fat weight to the body weight.

### 2.2 Glucose tolerance test and insulin tolerance test

For glucose tolerance test, rats were fasted overnight and administrated with glucose (2 g/kg) by gavage. For insulin tolerance test, rats were fasted for 6h and intraperitoneally injected insulin (1 IU/kg). Blood glucose contents at 0 min, 15 min, 30 min, 60 min, 90 min, and 120 min were recorded and blood glucose area under curve (AUC) was calculated.

### 2.3 Blood pressure measurement

At the end of 12-week feeding, rats were anesthetized with pentobarbital and blood pressure were measured by carotid artery cannulation and monitored with the BL-420 system. At the end of the experiment, rats were sacrificed by injection of an overdose pentobarbital.

### 2.4 Morphological examination of adipose tissue

Isolated epididymal adipose tissue was fixed with 4% paraformaldehyde, dehydrated with gradient alcohol and then embedded in paraffin. Embedded wax block was sliced and epididymal sections were stained with hematoxylin for 20 min and with eosin for 15 min, respectively. After sealing slide with neutral gum, the morphology changes of adipocytes were visualized using optical microscope (Olympus, Japan) and the cross-sectional area of adipocytes were calculated with Image J software.

### 2.5 Quantitative real time RT-PCR

Total mRNA of epididymal adipose tissue was extracted with Trizol (Takara Bio Inc., Japan). The concentration and purity of extracted total mRNA were measured using NANODROP ONE (Thermo Scientific, USA). cDNA was synthesized with the PrimeScript^™^RT reagent kit (Takara Bio Inc., Japan) following the manufacturers’ instructions. Relative quantitative PCR was conducted with a TB Green Primer Ex Taq II (Takara Bio Inc., Japan) using LightCycler^®^ 96 Real-Time PCR System (Roche, USA). The primer sequences were obtained from Takara and listed in Table 2. Gene expressions were normalized with β-actin. Statistically relative quantification was analyzed with equation 2^-ΔΔCT^. Ct is the threshold cycle to detect fluorescence.

**Table 2:** Primer sequences used for quantitative real time RT-PCR.

Name	Forward Primer (5’-3’)	Reverse Primer (5’-3’)
FAS	ACCTCATCACTAGAAGCCACCAG	GTGGTACTTGGCCTTGGGTTTA
ACC	TACAACGCAGGCATCAGAAG	TGTGCTGCAGGAAGATTGAC
CPT1a	CTGCCAGTTCCATTAAGCCACA	CAGCTATGCAGCCTTTGACTACCA
PPARα	GGCAATGCACTGAACATCGAG	GCCGAATAGTTCGCCGAAAG
PPARδ	TGGCCCTGTTCCTAGAATTGATG	GCAAACTCTGCCTGTGAGCTG
Dio2	CTGTGGTTGGATGTAGTCACACGA	CTTTGCACCAGGACCCAAATG
β-actin	ACGGTCAGGTCATCACTATCG	GGCATAGAGGTCTTTACGGATG
Nox4	ACTGGTGAAGATTTGCCTGGAAG	CACAGTATAGGCACAAAGGTCCAGA
Agtr1a	CCCACTCAAGCCTGTCTACGAA	GTGTGCTTTGAACCTGTCACTCC
Agtr1b	TCACACGCAGGCTTGTCAAC	CACTTCACTTGCAGGCTTTGAAC
UCP1	TGTGCAATGACCATGTACACCAA	GCACACAAACATGATGACGTTCC
PGC-1α	GCACTGACAGATGGAGACGTGA	TCATTGTAGCTGAGCTGAGTGTTGG
CD36	AACCCAATGGAGCCATCTTTGA	GTTGAGCACACCTTGAACAAATGAG
ACOX1	GGCCGCTATGATGGAAATGTG	GGGCTTCAAGTGCTTGTGGTAA

### 2.6 Measurement of free fatty acids and glycerol release in adipose tissue

At the end of 12-week feeding, rats were anesthetized with pentobarbital and sterilized with alcohol. Epididymal adipose tissue was isolated immediately and 200 mg adipose tissue was weight and cut into pieces, and then incubated in serum-free DMEM for 24 hours. DMEM was collected to measure NEFAs and glycerol contents in the supernatant with commercial Kits following the manufacturer’s instructions.

### 2.7 Measurement of cAMP concentration

Epididymal adipose tissue supernatants were obtained by homogenate and centrifugation. Cyclic AMP in the supernatants were measured using cAMP assay kit (4339, Cell Signaling Technology) according to the manufacturer’s protocol.

### 2.8 Western blot analysis

Epididymal adipose tissue was homogenized with a tissue grinder at 4°C temperature and the lysate were centrifugation at 12000 rpm for 20 minutes. The protein concentrations were measured with BCA kit (KeyGEN BioTECH Corp., Ltd, Nanjin, China). The protein samples were denatured, separated by SDS-PAGE and then transferred to the PVDF membrane. Membranes were blocked with 5% skim milk or bovine serum albumin (BSA) for 3 hours, and then incubated overnight at 4°C with specific primary antibodies as follows: ATGL (2138S, Cell Signaling Technology), P-HSL ^(ser660)^ (4126S, Cell Signaling Technology), HSL (4107S, Cell Signaling Technology), P-GSK-3β ^(ser9)^ (5558S, Cell Signaling Technology), GSK-3β (9315S, Cell Signaling Technology), β-actin (AP0060, Bioworld), PKA (5842S, Cell Signaling Technology), P-p38 ^(Thr180/tyr182)^ (9219S, Cell Signaling Technology), p38 (ab170099, Abcam), P44/42 MAPK (ERK1/2) (9102S, Cell Signaling Technology), P-P44/42 MAPK (ERK1/2) ^(Thr202/Tyr204)^ (Cell Signaling Technology) and AT2R (ab92445, Abcam). Membranes were washed and incubated with secondary antibodies (BA1054, BOSTER Biological Technology) at room temperature for 3 hours. After washing, membranes were exposed by Super ECL Prime (SEVEN BIOTECH) with ChemiDoc^™^ Imaging System (BIO-RAD, USA). The images were analyzed quantitatively by densitometry with Image J software.

### 2.9. Statistical analysis

Results were shown as mean ± SEM. Data were analyzed by two-way ANOVA to test for differences on the same diet within each genotype or differences in the WT or AT1aR^-/-^ rats on different diet. A two-way repeated ANOVA was used for comparisons over time. GraphPad Prism 6 was used for statistical analysis. A value of *p* < 0.05 was considered statistically significant.

## 3. Results

### 3.1 AT1aR knockout improved insulin sensitivity and metabolic disorders in high-fat diet rats

**Fig 1:**
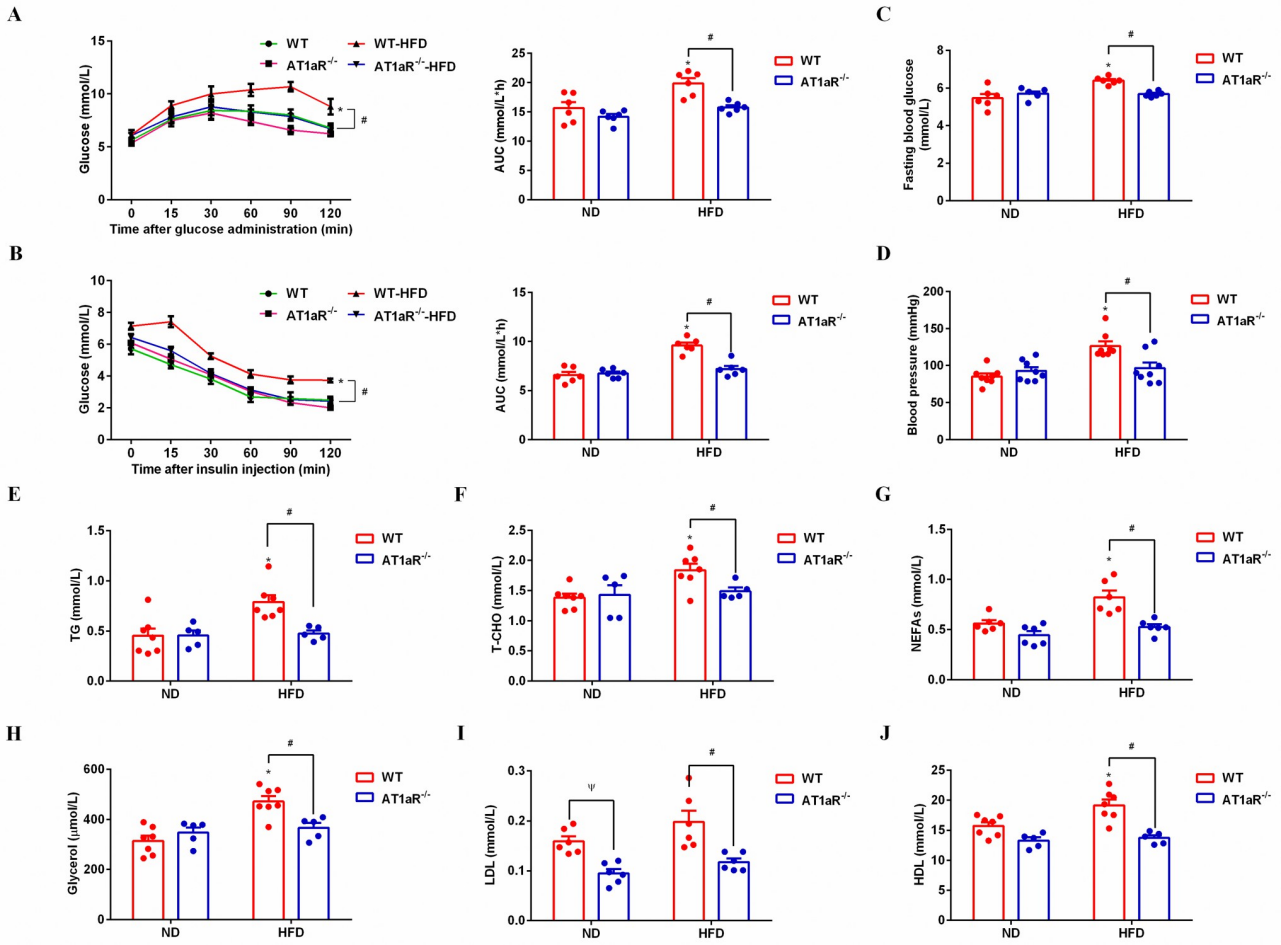
AT1aR knockout improved insulin resistance and metabolic disorders in high-fat diet rats. A: Oral glucose tolerance test (OGTT) curve and area under the curve. B: Insulin tolerance test (ITT) curve and area under the curve. C: Fasting blood glucose (FBG). D: Blood pressure. E: Serum triglyceride (TG) levels. F: Serum total cholesterol (T-CHO). G: Serum free fatty acids (NEFAs). H: Serum glycerol. I: Serum low-density lipoprotein (LDL). J: Serum high-density lipoprotein (HDL). *p < 0.05 vs WT-ND rats; ^#^*p* < 0.05 vs WT-HFD rats. Data were presented as Mean ± S.E.M. n = 6.

Glucose tolerance test and insulin tolerance test are important indicators that generally to be used to measure glucose tolerance and insulin sensitivity, respectively [18]. In response to glucose load or insulin injection, there was no significant differences between AT1aR^-/-^ rats and WT rats with normal diet, while the area under the curve (AUC) in HFD-fed AT1aR^-/-^ rats was significantly lower than that in the HFD-fed WT rats (Fig 1A and 1B), indicating that AT1aR knockout improved insulin sensitivity in high-fat-diet rats. Consistent with this, AT1aR knockout reduced the fasting blood glucose compared with WT rats fed with high-fat-diet (Fig 1C). Moreover, rats blood pressure was monitored through carotid artery intubation and the result showed that AT1aR gene knockout could reduce hypertension caused by high-fat feeding (Fig 1D).

Next, lipid metabolism-related indicators in serum were detected with commercial kits. The results showed that serum levels of TG, T-CHO, NEFAs, glycerol, LDL and HDL in high-fat diet WT rats were significantly higher than those of WT rats fed with normal diet, whereas these alterations were significantly reversed by AT1aR gene knockout, demonstrating that AT1aR knockout improved metabolic disorders in obese rats (Fig 1E–1J). Besides, serum LDL level of AT1aR^-/-^ rats were also lower than that of the WT rats fed with normal diet (Fig 1I).

### 3.2 AT1aR knockout alleviated high-fat diet-induced adipocyte hypertrophy

**Fig 2:**
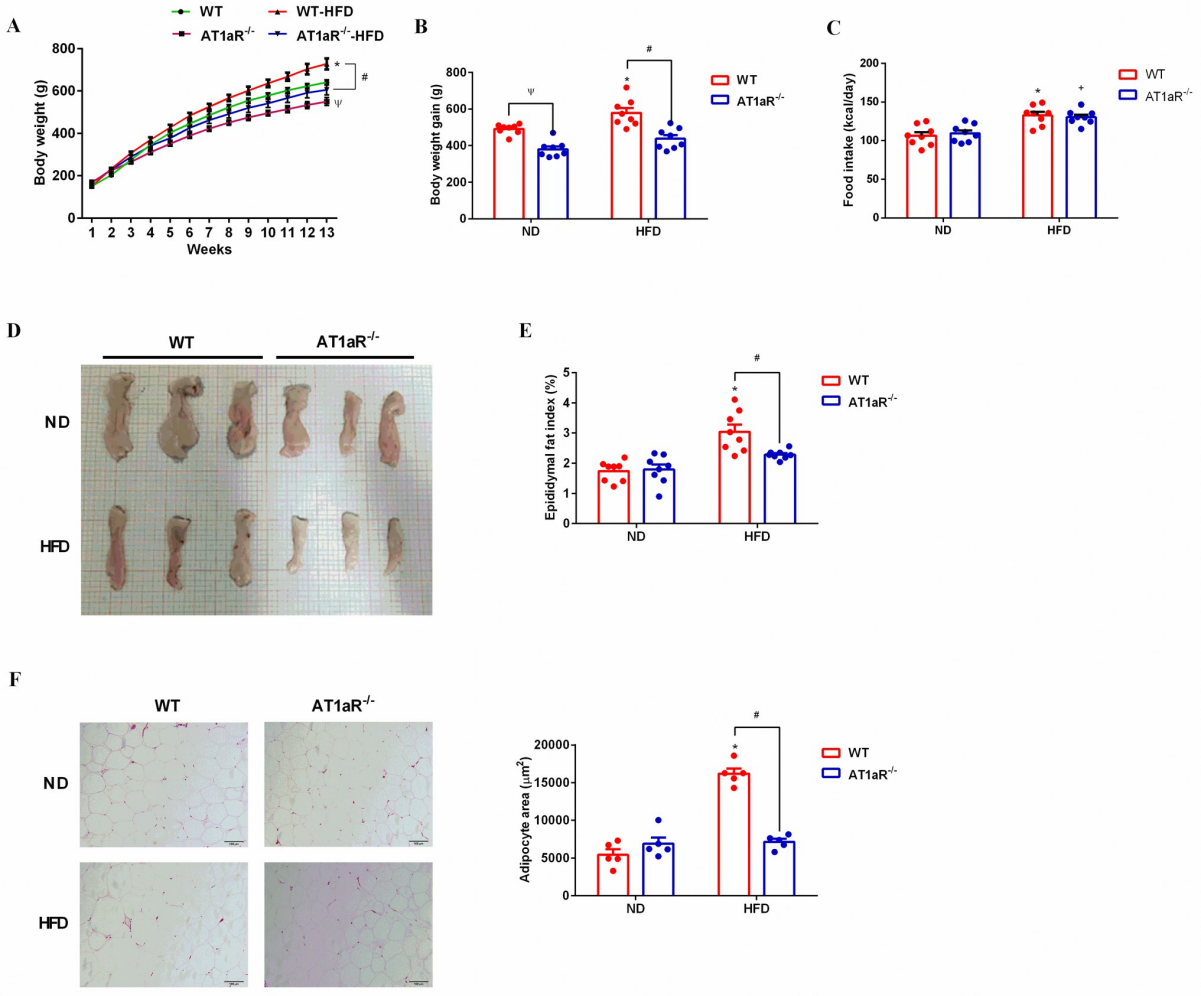
AT1aR knockout improved epididymal histomorphology induced by high fat diet. A, B: Body weight of rats was recorded weekly and body weight gain was calculated by weight increase in 12 weeks. C. Daily food intake of rats. D: Physical morphology of epididymal fat. E: Epididymal fat index (showed as epididymal fat weight/body weight). F: Adipocytes morphology was visualized by HE staining and adipocyte area were calculated by Image J. *p < 0.05 vs WT-ND rats; ^+^*p* < 0.05 vs AT1aR^-/—^ND rats; ^Ψ^*p* < 0.05 vs WT-ND rats. Data were presented as Mean ± S.E.M. n = 6.

Body weight and food intake of rats were recorded weekly and the results showed that body weight gain of WT rats were increased by high-fat diet feeding, while body weight gain of high-fat diet AT1aR^-/-^ rats were much lower than that of high-fat diet WT rats (Fig 2A and 2B). And the average calorie intake was unchanged between WT and AT1aR^-/-^ rats either with normal diet or high-fat diet (Fig 2C). Epididymal adipose tissues were isolated and their physical morphology were photographed. As showed in Fig 2C, physical morphology of epididymal adipose tissue in AT1aR^-/-^ rats was smaller than that of WT rats. Besides, the epididymal fat index of high-fat diet AT1aR^-/-^ rats was also decreased compared to high-fat diet WT rats (Fig 2D). Moreover, the histomorphology of adipose were visualized by HE staining and adipocyte area of WT rats were significantly enlarged by high-fat diet feeding, whereas this alteration were significantly reversed in high-fat diet AT1aR^-/-^ rats (Fig 2E). These results indicated that AT1aR gene knockout improved adipose histomorphology and inhibited adipocyte hypertrophy.

### 3.3 AT1aR gene knockout promoted adipose lipolysis

**Fig 3:**
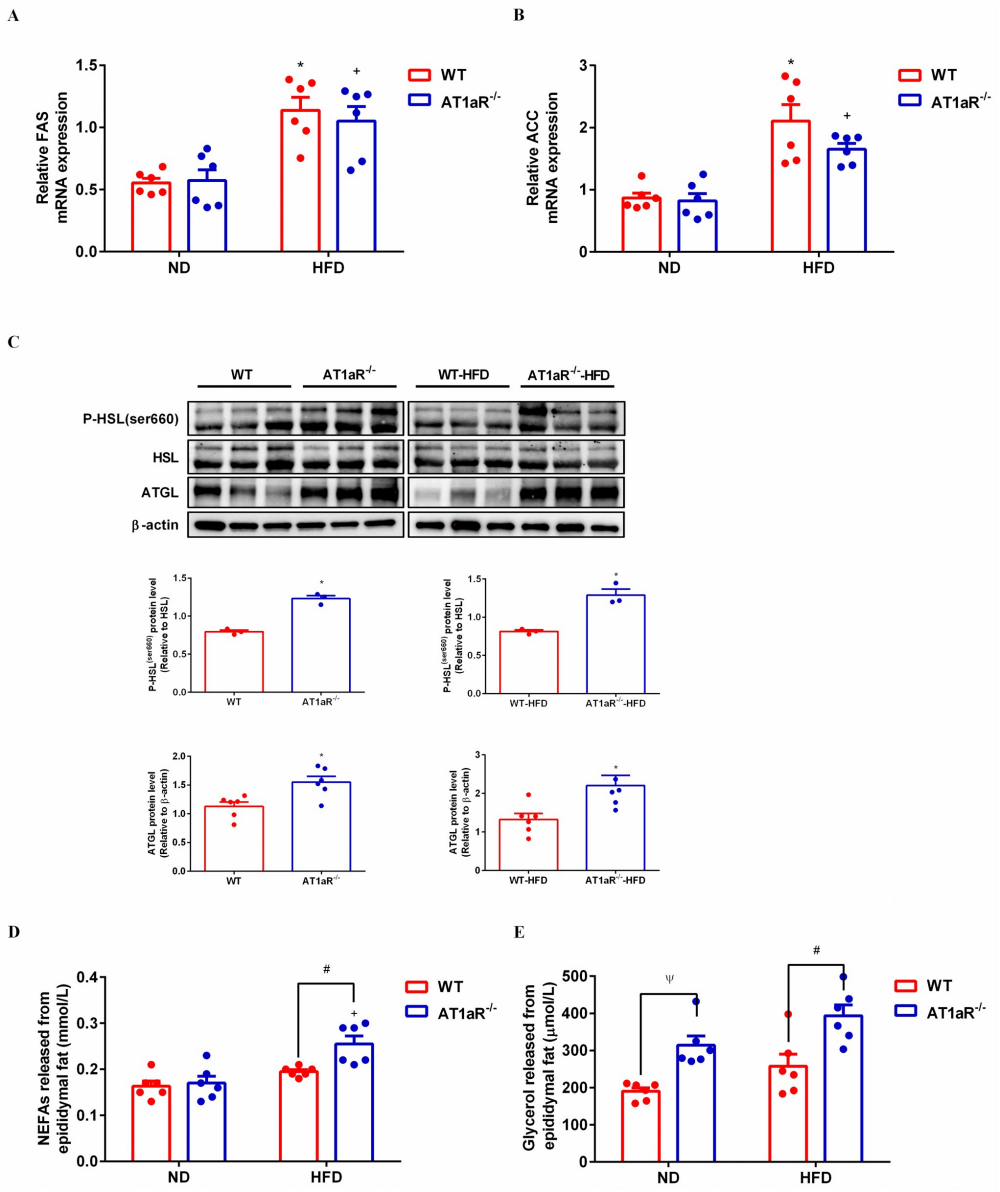
AT1aR knockout promoted adipose lipolysis. A, B: Gene expressions of key enzymes for lipid synthesis in adipose tissue, FAS (A) and ACC (B). C: Protein expressions of adipose lipolysis related enzymes, ATGL and HSL. D, E: Levels of NEFAs (D) and glycerol (E) in culture medium released by adipose tissue within 24 hours. *p < 0.05 vs WT-ND rats; ^+^*p* < 0.05 vs AT1aR^-/—^ND rats. Data were presented as Mean ± S.E.M. n = 6.

The main function of white adipose tissue is to store energy in the form of triglycerides and the fat mass is systematically regulated by lipid synthesis and decomposition. In order to know the underlining mechanism by which AT1aR gene knockout inhibited adipocyte hypertrophy, we detected the expressions of major enzymes for triglyceride synthesis and lipolysis in the adipose tissue. Fatty acid synthase (FAS) and acetyl-CoA carboxylase (ACC) are rate-limiting enzymes involved in biosynthesis of fatty acids, an important step of lipogenesis [19]. Gene expressions of FAS and ACC were measured with RT-PCR and the results showed that there were no significant differences between WT and AT1aR^-/-^ rats neither fed with normal diet nor with high-fat diet (Fig 3A and 3B). The hydrolysis of triglycerides is initiated by adipose triglyceride lipase (ATGL) [20], and hormone sensitive lipase (HSL) is the main hydrolase for triacylglycerol (TAG) and diacylglycerol (DAG) [21]. As showed in Fig 3C, protein expression of ATGL and phosphorylation of HSL in AT1aR^-/-^ rats were much higher than those of WT rats both in the normal and high-fat diet rats. Furthermore, the fresh adipose tissue was cultured in the DMEM for 24 hours and the levels of NEFAs and glycerol in the culture medium were detected. Compared with WT rats fed with high-fat diet, the levels of NEFAs and glycerol released from adipose tissue of high-fat diet AT1aR^-/-^ rats increased significantly (Fig 3D and 3E). These results proved that AT1aR knockout improved adipose histomorphology in high-fat diet rats by promoting adipose lipolysis, while did not affect lipid synthesis.

### 3.4 AT1aR knockout accelerated adipose energy expenditure and fatty acids oxidation

**Fig 4:**
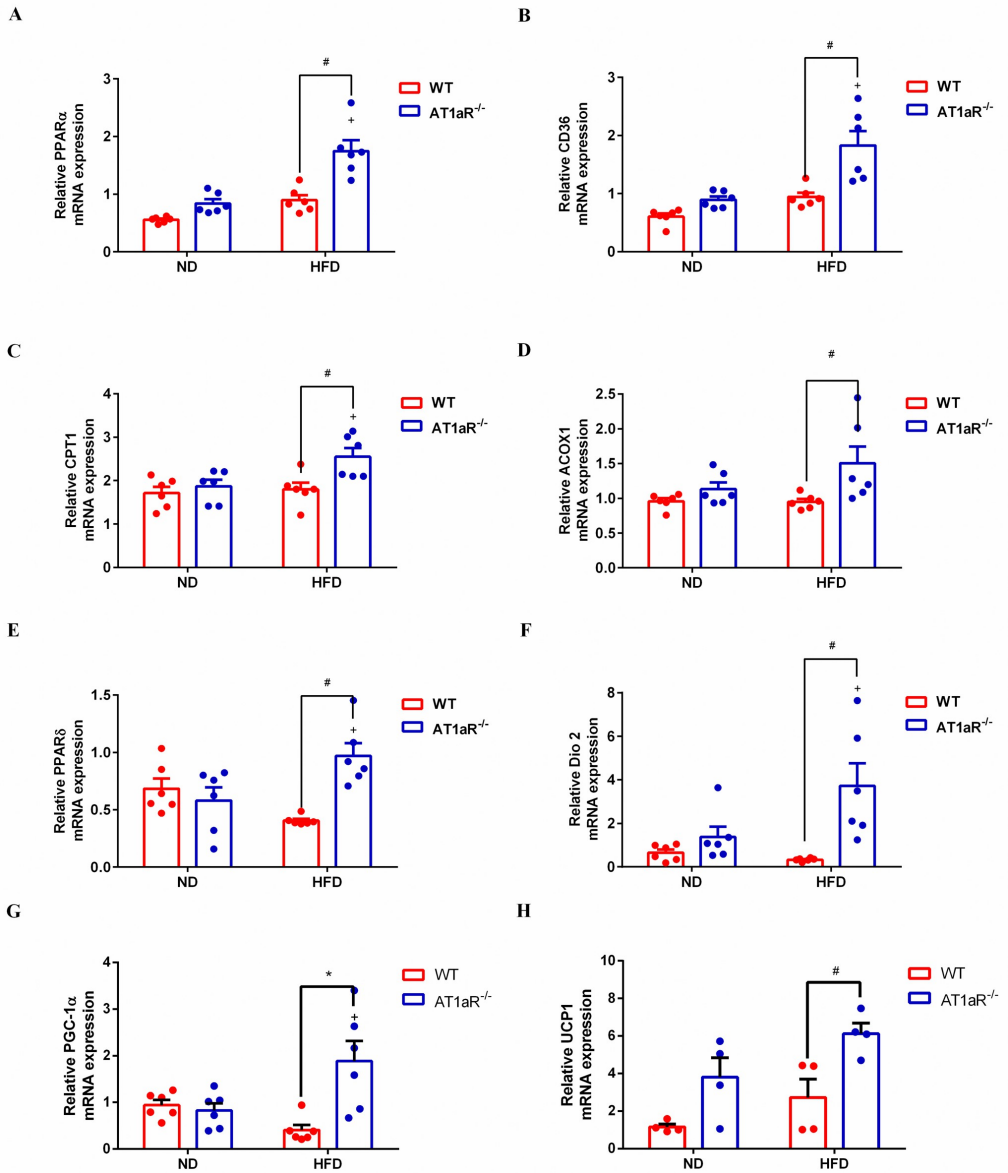
AT1aR knockout elevated adipose energy expenditure and fatty acid oxidation. A-D: Gene expressions of PPARα (A), CD36 (B), CPT1 (C) and ACOX1 (D) in adipose tissue. E-H: Gene expressions of PPARδ (E), Dio2 (F), PGC-1α (G) and UCP1 (H) in adipose tissue. ^+^*p* < 0.05 vs AT1aR^-/—^ND rats. Data were presented as Mean ± S.E.M. n = 6.

Subsequently, adipose lipid utilization and energy expenditure were detected. Peroxisome proliferator-activated receptors α (PPARα) acts as a transcription factor to regulate a series of genes involved in fatty acid uptake such as fatty acid translocase CD36 and mitochondrial fatty acid oxidation like carnitine palmitoyl transferase 1 (CPT1), acyl-coenzyme A oxidase 1 (ACOX1) [22, 23]. The results showed that the gene expressions of PPARα, CD36, CPT1 and ACOX1 were significantly increased in AT1aR^-/-^ rats compared with WT rats fed with high-fat diet. However, there were no differences between the WT and AT1aR^-/-^ rats fed a normal diet (Fig 4A–4D). These results indicated enhanced fatty acid oxidation in high-fat diet AT1aR^-/-^ rats. Besides, energy expenditure in adipose tissue was also measured. Peroxisome proliferator activated receptor δ (PPARδ) is a transcription factor that promote oxidation in adipose [24] and deiodinase 2 (Dio2) could activate thyroid hormone to promote energy expenditure [25]. Peroxisome proliferator-activated receptor γ coactivator 1α (PGC-1α) is a key regulator of mitochondria biogenesis and global oxidative metabolism [26]. Adipocytes express uncoupling protein-1 (UCP-1) to dissipate heat instead of generating ATP [27], and thus reflecting the thermogenesis and energy expenditure in adipose tissue. By comparison of WT rats fed with high-fat diet, the results showed that the gene expressions of PPARδ, Dio2, PGC-1α and UCP1 in adipose tissue were significantly increased in high-fat diet AT1aR^-/-^ rats (Fig 4E–4H), indicating increased energy consumption in adipose tissue of AT1aR^-/-^ rats.

### 3.5 High-fat diet activated Ang II-AT1R axis in rats

**Fig 5:**
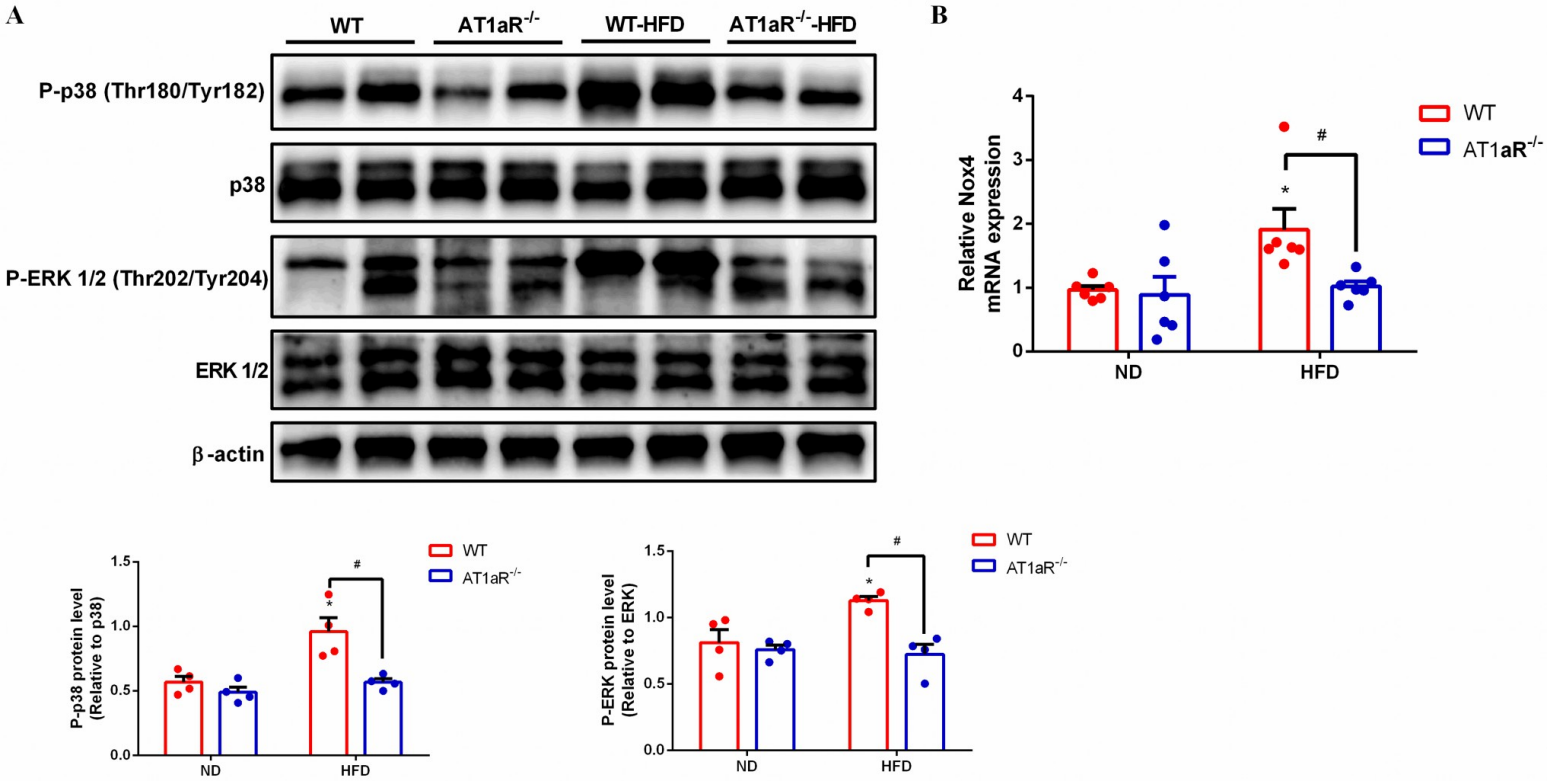
High fat diet activated Ang II-AT1R axis in rats. A. Protein expressions of kinases downstream of the Ang II-AT1R axis, p38 and ERK. B. Gene expression of Nox4 in adipose tissue. *p < 0.05 vs WT-ND rats. Data were presented as Mean ± S.E.M. n = 4.

RAS over-activation is a common characteristic in obesity patients, but considering that high fat diet is not a direct stimulator for the RAS. To confirm the activation of Ang II-AT1R axis in high-fat diet rats, the down-stream pathway of the axis: nicotinamide adenine dinucleotide phosphate oxidases (NADPH oxidases) and phospho-p38 mitogen-activated protein kinases (MAPKs) were examined. P38 MAPK and the extracellular signal-regulated kinases 1/2 (ERK 1/2) are subfamilies of MAPK signaling pathway [28]. The result showed that the phosphorylation of P38 and ERK 1/2 were obviously elevated in WT rats fed with high-fat diet than those with normal diet. However, the increased phosphorylation of P38 and ERK 1/2 in high-fat diet-WT rats were significantly reversed by AT1aR deficiency (Fig 5A). Consistent with this, the gene expression of NADPH oxidases (NOX4), the major isoform in adipocyte [29], was increased in high-fat diet-WT rats and reversed by AT1aR deficiency (Fig 5B). Collectively, these results suggesting that high fat diet actually activated Ang II-AT1R axis and AT1aR deficiency could reverse these alterations.

Besides, the gene expression of AT1bR and AT2R protein expression in adipose tissue were also detected and the results showed that there were no statistically differences between WT and AT1aR deficiency rats (S2 Fig).

### 3.6 AT1aR knockout activated cAMP/PKA pathway

**Fig 6:**
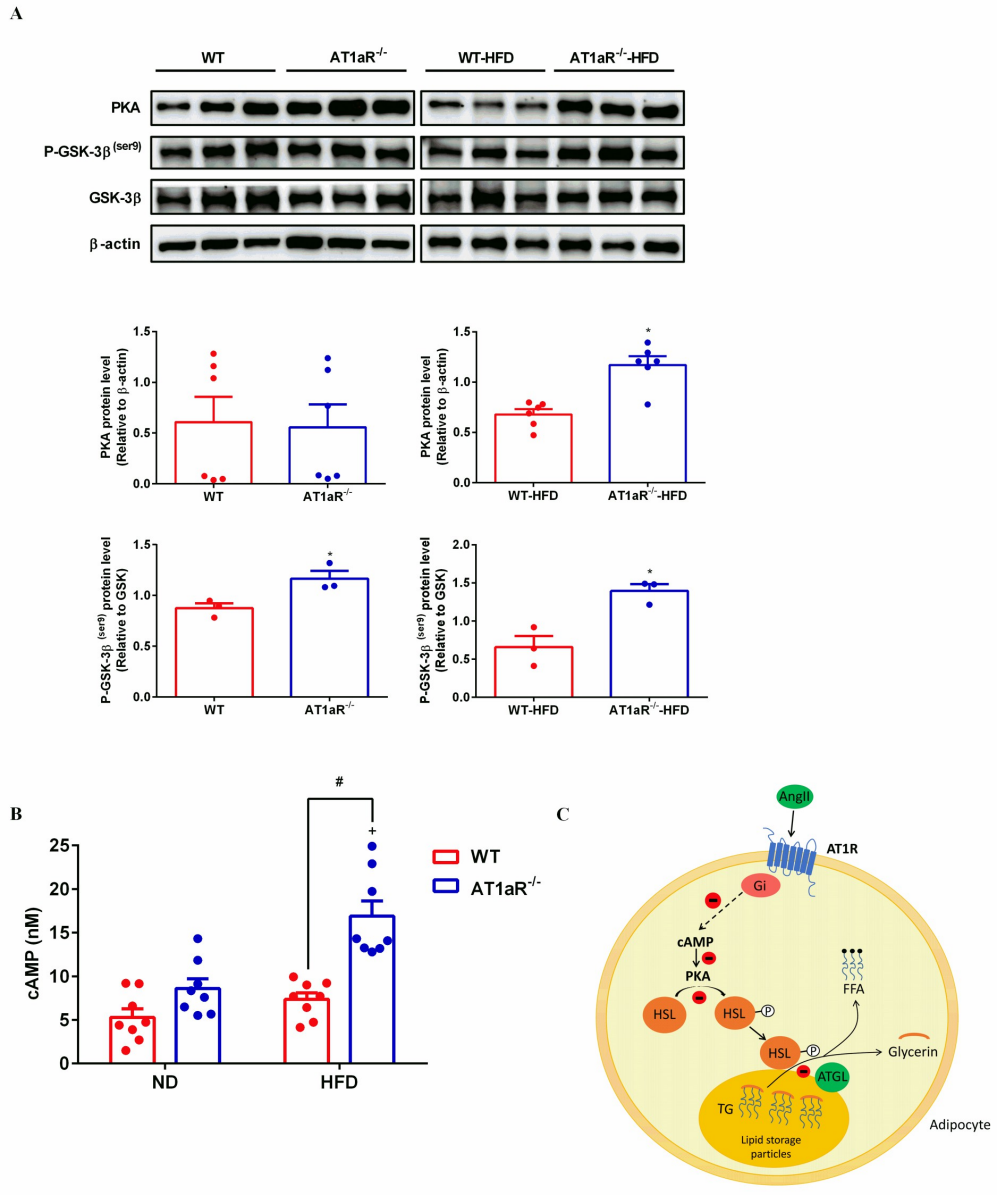
AT1aR knockout activated cAMP/PKA pathway. A: Protein expressions of PKA, P-GSK-3β (ser9) and GSK-3β in adipose tissue. B: cAMP levels in adipose tissue. C: The proposed pathway for Ang II inhibiting adipose lipolysis. By binding to AT1R, Ang II activates inhibitory Gi which in turn reduces cAMP production. As a result, PKA activation is limited and HSL phosphorylation is decreased, leading to inhibitory of triglyceride hydrolysis. ^+^*p* < 0.05 vs AT1aR^-/—^ND rats. Data were presented as Mean ± S.E.M. n = 6.

It has been reported that the binding of Ang II to AT1R activates Gi protein against the effect of cAMP/PKA pathway [30]. As showed in Fig 6A, PKA levels were significantly increased in AT1aR^-/-^ rats fed with high-fat. Consistently, cAMP levels in WT rats were not changed fed with high-fat diets, while obviously elevated in high-fat diet AT1aR^-/-^ rats (Fig 6B). Protein kinase A (PKA) that activated by cyclic adenosine monophosphate (cAMP) mainly phosphorylates HSL to mediate adipose lipolysis [31]. Apart from HSL, GSK-3β is also an important phosphorylation substrate of PKA [32]. The phosphorylation levels of GSK-3β in AT1aR^-/-^ rats were higher than WT rats both fed with normal diet and high-fat diet (Fig 6A), further confirmed the enhancement of PKA activity in AT1aR^-/-^ rats. These results suggested that gene knockout of AT1aR could activate cAMP/PKA pathway.

## 4. Discussion

Obesity is the common pathological basis of many metabolic diseases and its mechanism exploration has attracted more and more attention. RAS is generally considered acting in the regulation of blood pressure and organism water-salt balance. However, clinical studies have demonstrated that type 2 diabetes can be delayed by treatment with ACEI and ARB compared with other antihypertensive drugs [33]. Besides, captopril, one of ACEI drugs, was showed to not only lower blood pressure but also reduce the weight of high-fat diet rats [34]. These studies indicated that RAS plays an important role in obesity and subsequent metabolic diseases, whereas the regulatory mechanism remains unclear. In the present study, we found that the gene knockout of AT1aR improved high-fat diet induced obesity by promoting lipolysis through cAMP/PKA pathway, providing new sight for the prevention and treatment of obesity and related metabolic disorders.

As the primary peptide of the RAS, Ang II exerts its biological activity mainly through AT1R in adipose tissue. Different from human beings, AT1R is coded by two gene subtypes, AT1aR and AT1bR, in rodent mammals [35]. AT1aR has the most homology with human, mainly involved in the vasoconstriction and blood pressure regulation. While AT1bR is related to the thirst response of mammals and existed in certain areas of the central nervous system and adrenal tissues [36]. So, in the present study, we constructed AT1aR^-/-^ rats to explore the relation between RAS and obesity. As expected, AT1aR^-/-^ rats lacked mRNA expression of a complete AT1aR in major Ang II responsive tissues, while AT1bR gene expression and AT2R protein expression in the adipose tissue were showed no differences between WT and AT1aR^-/-^ rats fed with normal diet or high-fat diet, demonstrating that AT1aR deficiency did not alter AT1bR and AT2R function in the adipose tissue.

Obesity is closely correlated with insulin resistance and diabetes. Besides storage energy, adipose tissue also acts as an endocrine organ. In obesity, adipocytes secrete inflammatory adipokines which damage insulin signaling pathway, resulting in disorders of glucose and lipid metabolism [37]. Here, we showed that AT1aR gene knockout improved glucose intolerance and insulin sensibility in high-fat diet induced obese rats. Consistent with our results, Ryuji Kouyama et al. showed that Ang II modulate adipocytokine production via AT1aR and AT1aR deficiency protected from high-fat diet-induced impairment of glucose tolerance and insulin resistance [17]. Besides, Kengo Azushima et al. has demonstrated that upregulation of AT1R-related protein (ATRAP), a protein limiting AT1R’s effects by promoting its internalization, could improve insulin resistance [18]. The results showed that AT1aR deficiency improved dyslipidemia induced by high-fat diet, while deletion of the AT1aR did not cause major metabolic alterations in AT1aR^-/-^ rats fed with a regular diet. In physiological condition, RAS were not over-activated. However, with excess calories intake in obesity condition, the RAS is over-activated indicated by increased activities of NADPH oxidases and MAPKs. In this context, AT1aR deficiency has a significantly positive role in maintaining metabolic homeostasis. Consistent with our study, AT1aR deficiency by systemically or specific in adipocytes do not influence metabolic parameters in mice fed with normal diet [17, 38].

Interestingly, no significant differences in systolic blood pressure were noted between WT rats and AT1aR^-/-^ rats fed with a standard diet in our present study, which was inconsistent with previous research [17]. In the present study, our results showed that AT1aR gene knockout in rat did not influence blood pressure with normal diet, while obviously reduced arterial blood pressure in rats with high-fat diet. Consistent with our results, previous study demonstrates that systolic blood pressure were unchanged between WT and AT1aR^-/-^ mice under normal condition, while systolic blood pressure were significantly reduced in AT1aR^-/-^ mice compared to WT mice after Ang II infusion [39]. The blood pressure homeostasis was maintained through multiple mechanisms and Ang II exert its major role in blood pressure regulation mainly under conditions that RAS were over-activated, such as decreased renal blood flow, hyponatremia and sympathetic activation from various causes. In physiological condition, RAS were not over-activated, and serum Ang II level in AT1aR gene knockout rat were equivalent with that in WT rat [40, 41]. So, it is reasonable that the AT1aR^-/-^ rats with normal diet were capable of maintaining blood pressure within normal range and blood pressure was not influenced obviously by AT1aR knockout. While the Ang II-AT1aR axis was over-activated in high fat diet rats, and AT1aR deficiency significantly reduced systolic blood pressure.

Lipid stocks in the adipose tissue fluctuate by lipid synthesis and lipolysis. Our results showed that AT1aR gene knockout improved adipocyte hypertrophy in obese rats mainly by promoting lipolysis. However, enhanced lipolysis increases circulatory NEFAs which leading to ectopic deposition of lipids. Interestingly, serum levels of NEFAs were decreased in high-fat diet AT1aR^-/-^ rats despite significant enhancement of lipolysis in adipose tissue. In this regard, energy expenditure and NEFAs oxidation in adipose tissue were measured. The results demonstrated that gene expressions of proteins involved in adipose tissue oxidative metabolism were elevated. Therefore, two main factors appear to contribute to the reduction of serum NEFAs level in HFD-fed AT1aR^-/-^ rats. First, AT1aR deficiency substantially reduced the mass of white adipose tissue, hence the net release of NEFAs from adipose tissue is likely lower than those be expected from AT1aR^-/-^ rats with elevated lipolytic rates. Second, despite increased lipolysis in adipose tissue of AT1aR^-/-^ rats, increased energy expenditure and enhanced fatty acids uptake and oxidation in adipose tissue may play a significant role in the reduction of serum NEFAs. In this context, AT1aR deficiency elevated lipolysis and promoted energy dissipation in white adipose tissue with decreased adipose mass, and was not necessarily increase circulating NEFAs levels. It seems that AT1aR deficiency increased lipolysis in adipose tissue may cause a shift within adipocytes towards increased fatty acid utilization and energy expenditure, which may make AT1aR as a target to regulate adipose lipolysis to protect against obesity.

The above results implicating a global metabolism improvement and energy expenditure enhancement in AT1aR deficiency rats. What’s more, the UCP1 expression in brown adipose tissue (BAT) was also measured and the result showed that AT1aR knockout significantly increased UCP1 mRNA expression in BAT, indicating an increased thermogenesis in AT1aR deficiency rats (S3 Fig). To verify an enhanced global expenditure, it is necessary to further investigate the locomotor activity and resting metabolic rate data to evaluate the overall energy expenditure. However, this result is lacking in our research and further studies focusing on BAT function will also be needed.

By binding to AT1R, a G protein-coupled receptor, Ang II activates inhibitory Gi protein which in turn decreases cAMP production [42, 43]. The cAMP/PKA pathway plays an important role in energy balance and metabolic regulation. As an important second messenger, increased cellular cAMP activates PKA which in turn phosphorylates HSL [44]. AT1aR gene knockout increased PKA protein expression and cellular levels of cAMP in the adipose tissue. Activated PKA can be confirmed by increased phosphorylation of GSK-3β, a main phosphorylation substrate of PKA [32].

In conclusion, our results showed that RAS predominant peptide Ang II promoted adipocyte hypertrophy by inhibition of cAMP/PKA and subsequent adipose lipolysis via binding to AT1aR. However, AT1aR gene knockout activated cAMP/PKA signaling and elevated adipose lipolysis by phosphorylation of HSL, and thus improving obesity and insulin resistance (Fig 5C). Our findings emphasized the special role of RAS signaling in adipose tissue in the progress of obesity and associated metabolic abnormalities, and provided potential drug target for their therapy.

## Supporting information

S1 Fig Development of AT1aR^-/-^ rats. A. sgRNA combined CRISPR/Cas system to generate AT1aR^-/-^ rats. B, C. PCR results for AT1aR^-/-^ rats and WT rats. M: marker; N: negative control; P: positive control D. AT1aR deficiency was confirmed in major Ang II responsive tissues. Data were presented as Mean ± S.E.M. n = 5.
(TIF)

S2 Fig AT1aR deficiency did not cause alterations of AT1bR and AT2R in adipose tissue. A. Gene expression of AT1bR in adipose tissue. B. Protein expression of AT2R in adipose tissue. Data were presented as Mean ± S.E.M. n = 4.
(TIF)

S3 Fig AT1aR knockout enhanced energy expenditure in brown adipose tissue. Gene expression of UCP1 in brown adipose tissue. Data were presented as Mean ± S.E.M. n = 5.
(TIF)

S1 Raw images (PDF)
