# Supplementary figures and images for: The gene knockout of angiotensin II type 1a receptor improves high-fat diet-induced obesity in rat via promoting adipose lipolysis
Source: PLoS One. 2022 Jul 8;17(7):e0267331. doi: 10.1371/journal.pone.0267331 (PMC9269876; doi:10.1371/journal.pone.0267331)

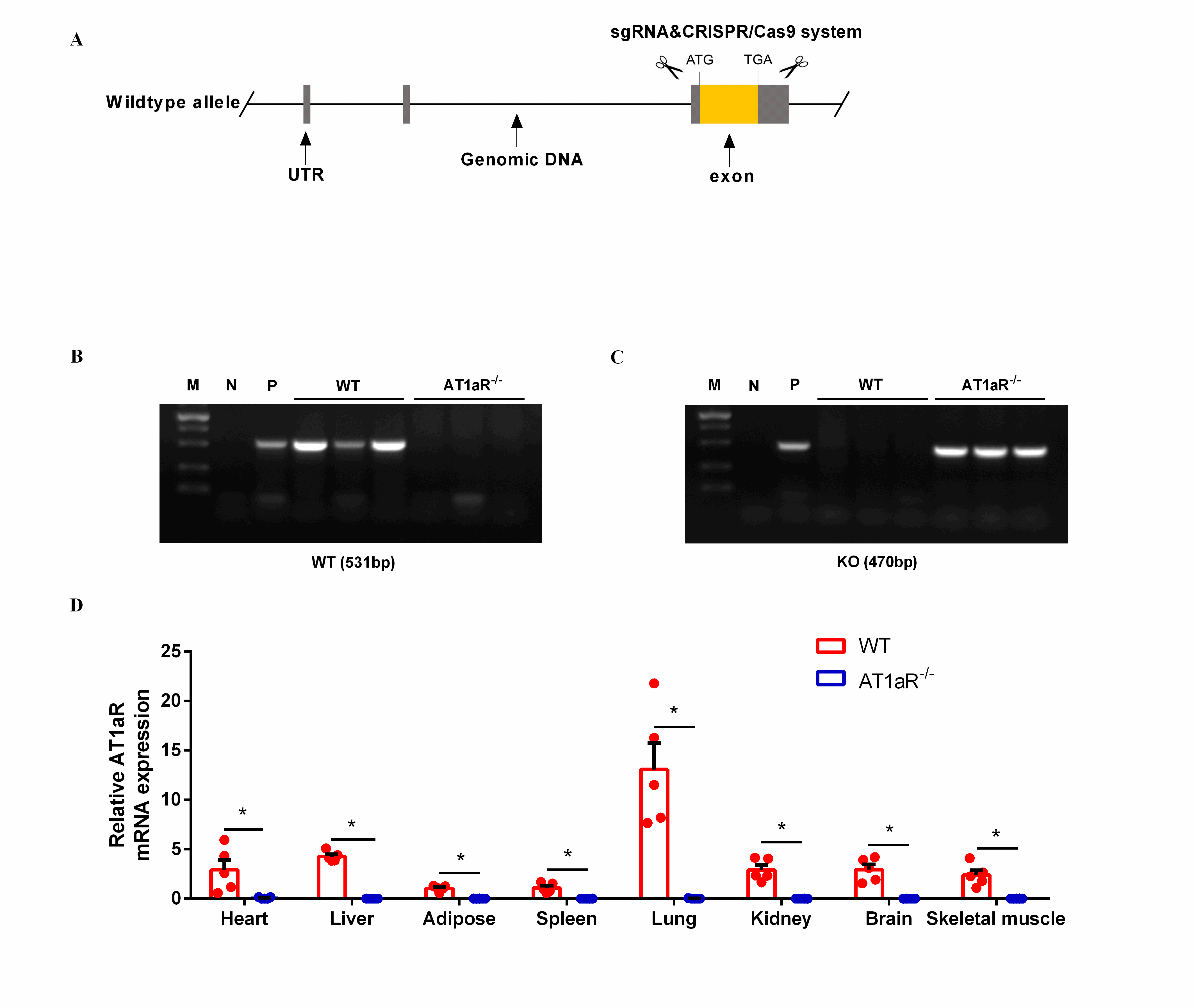

Supplement: S1 Fig — A. sgRNA combined CRISPR/Cas system to generate AT1aR-/- rats. B, C. PCR results for AT1aR-/- rats and WT rats. M: marker; N: negative control; P: positive control D. AT1aR deficiency was confirmed in major Ang II responsive tissues. Data were presented as Mean ± S.E.M. n = 5. (TIF) [file pone.0267331.s001.tif]

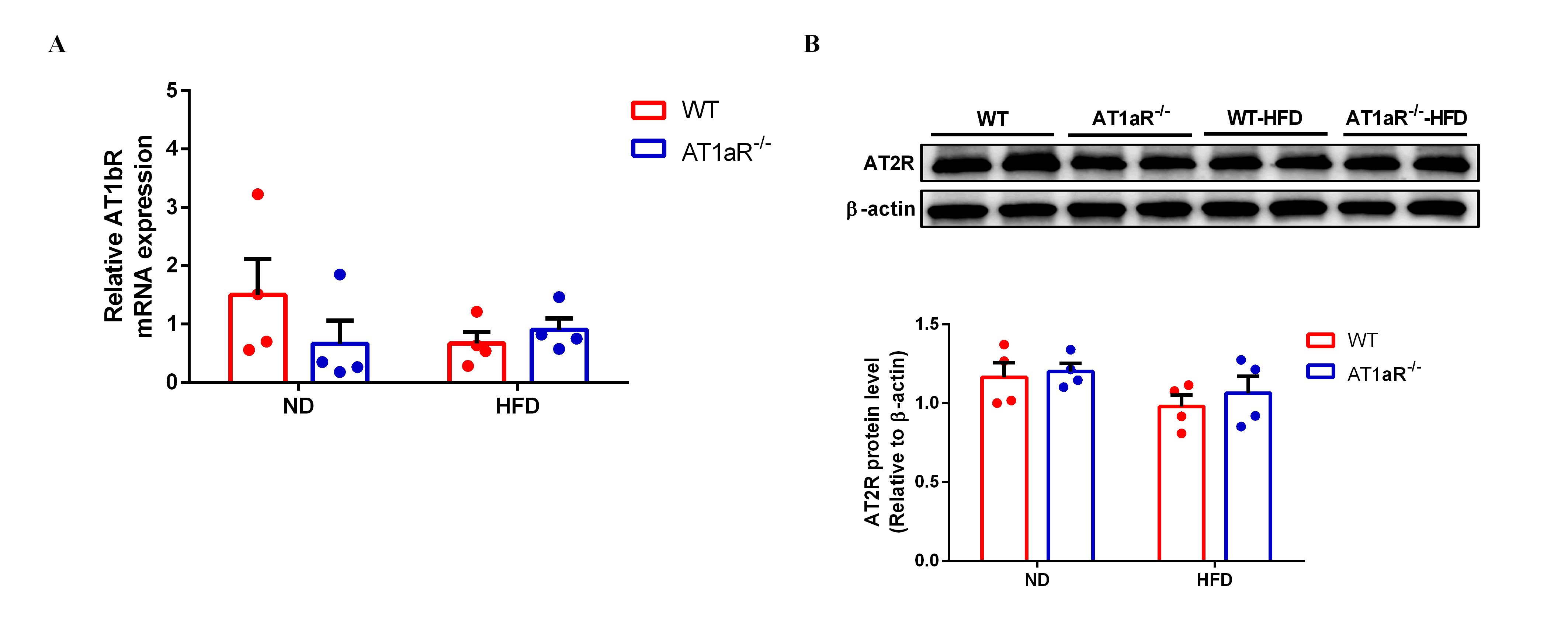

Supplement: S2 Fig — A. Gene expression of AT1bR in adipose tissue. B. Protein expression of AT2R in adipose tissue. Data were presented as Mean ± S.E.M. n = 4. (TIF) [file pone.0267331.s002.tif]

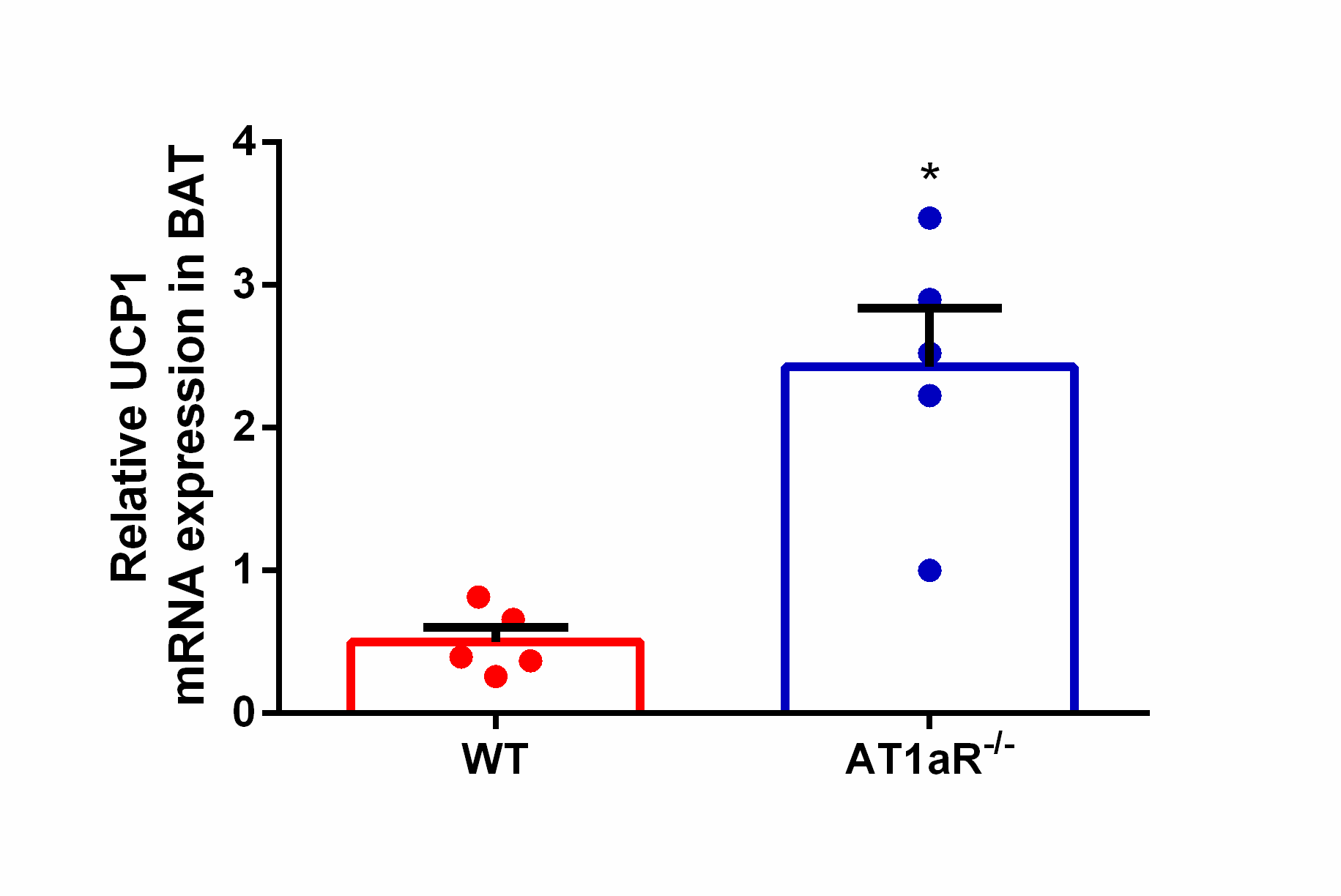

Supplement: S3 Fig — Gene expression of UCP1 in brown adipose tissue. Data were presented as Mean ± S.E.M. n = 5. (TIF) [file pone.0267331.s003.tif]

Raw images in Fig 3.

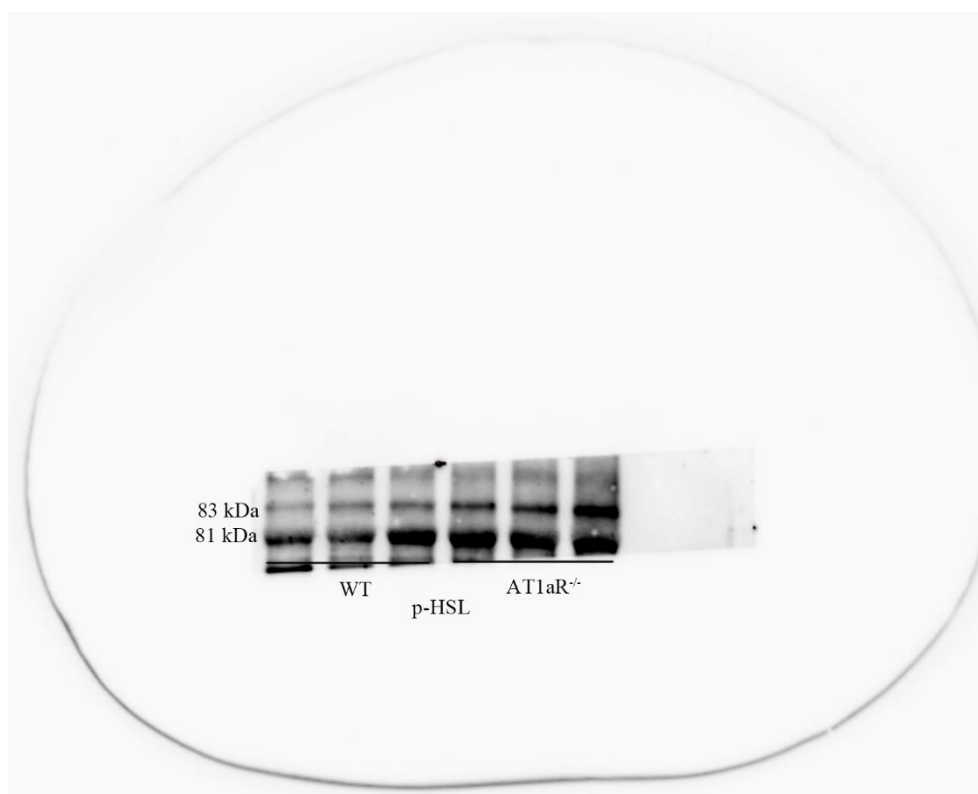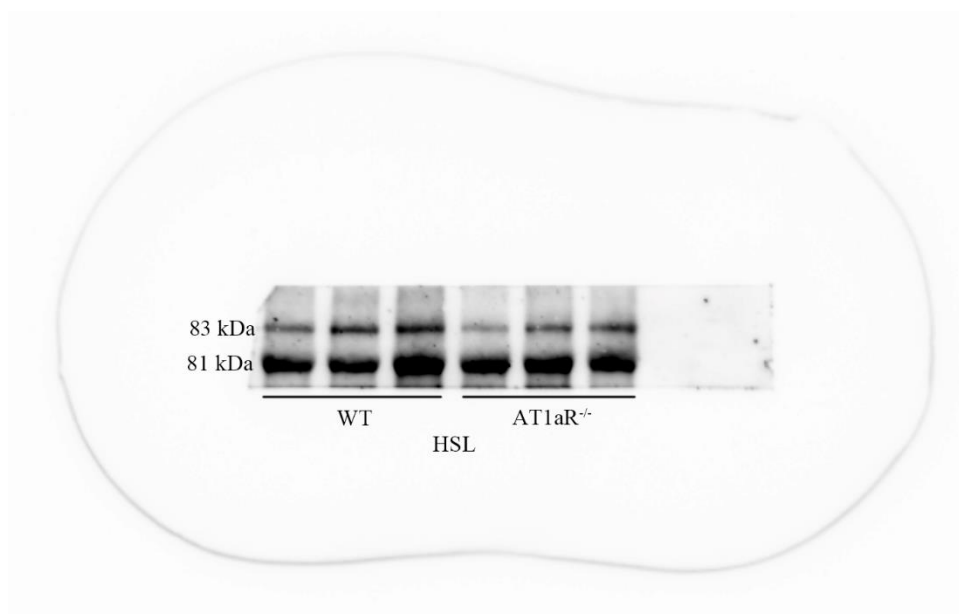

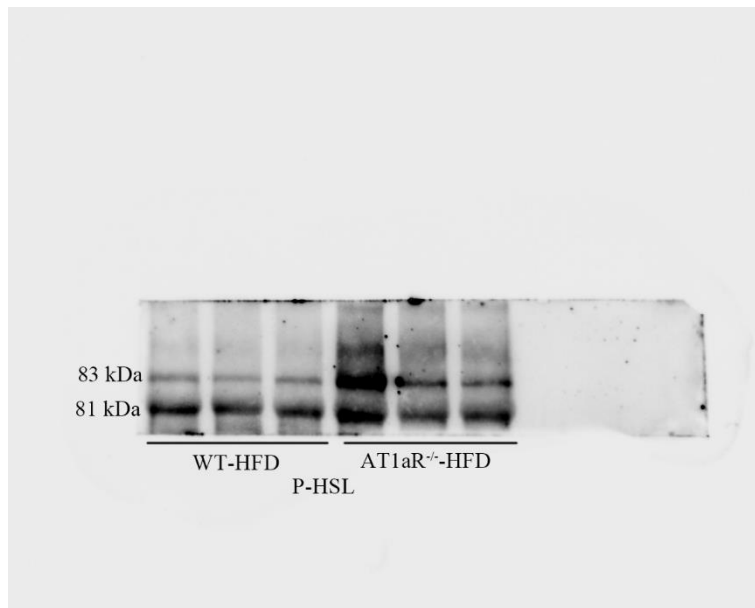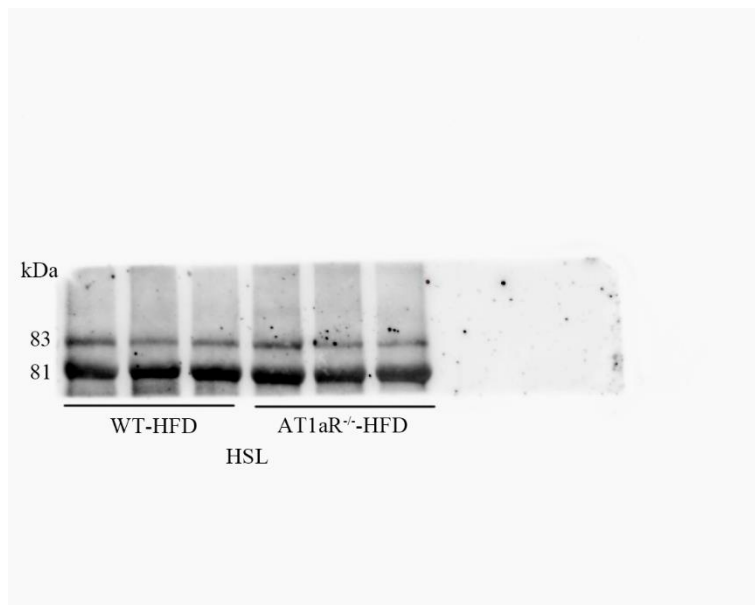

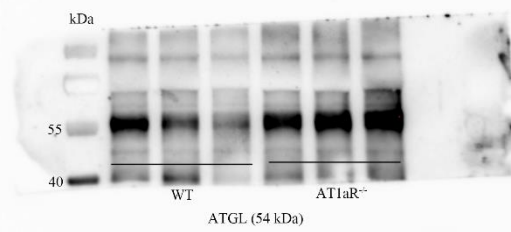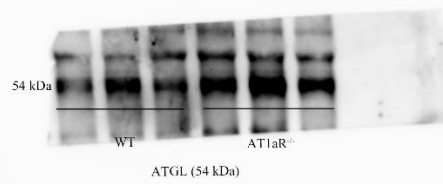

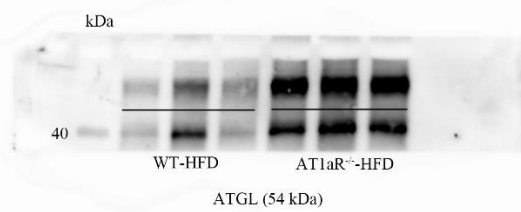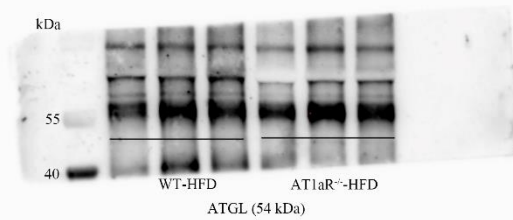

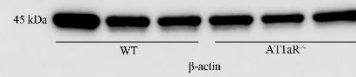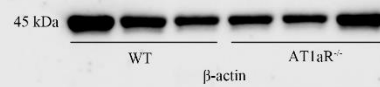

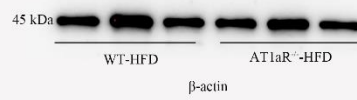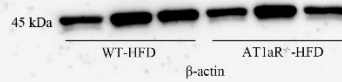

Raw images in Fig 5.

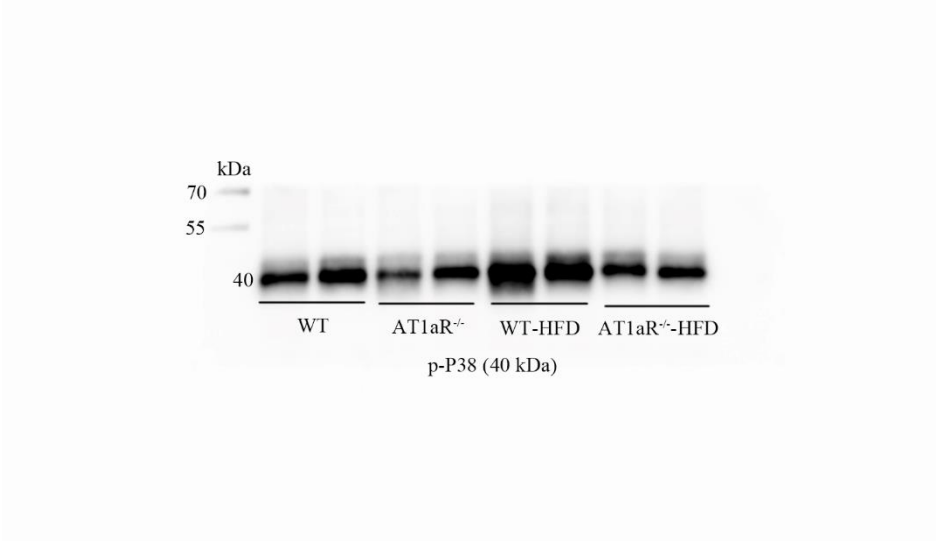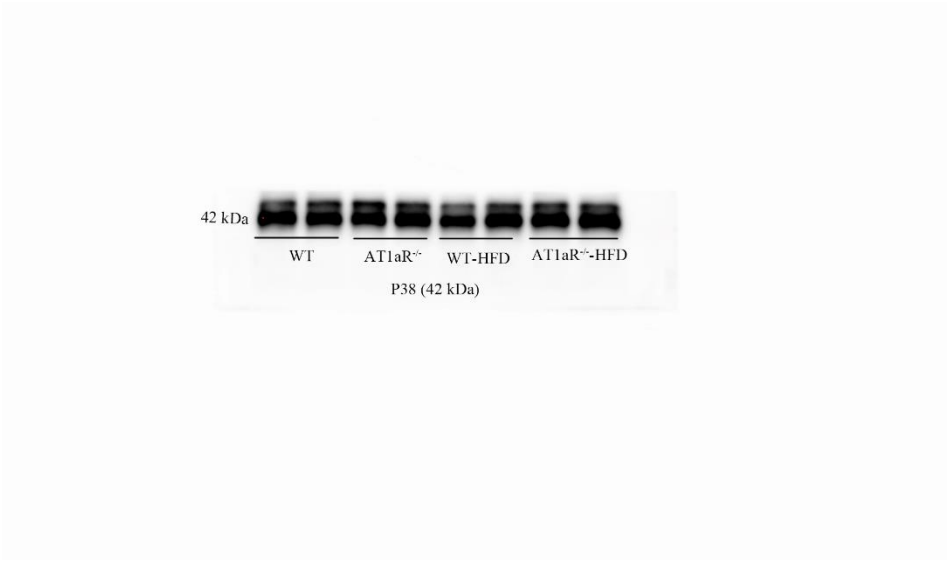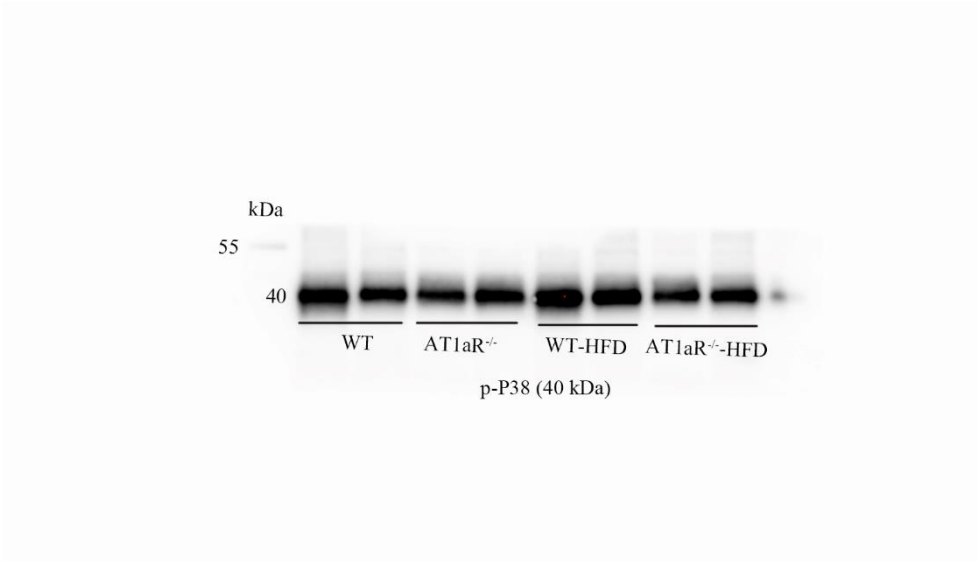

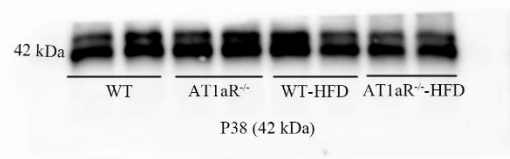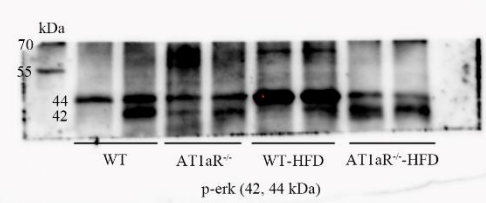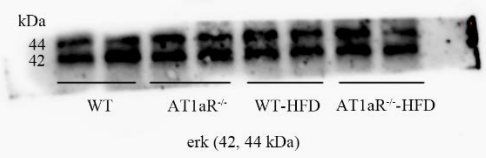

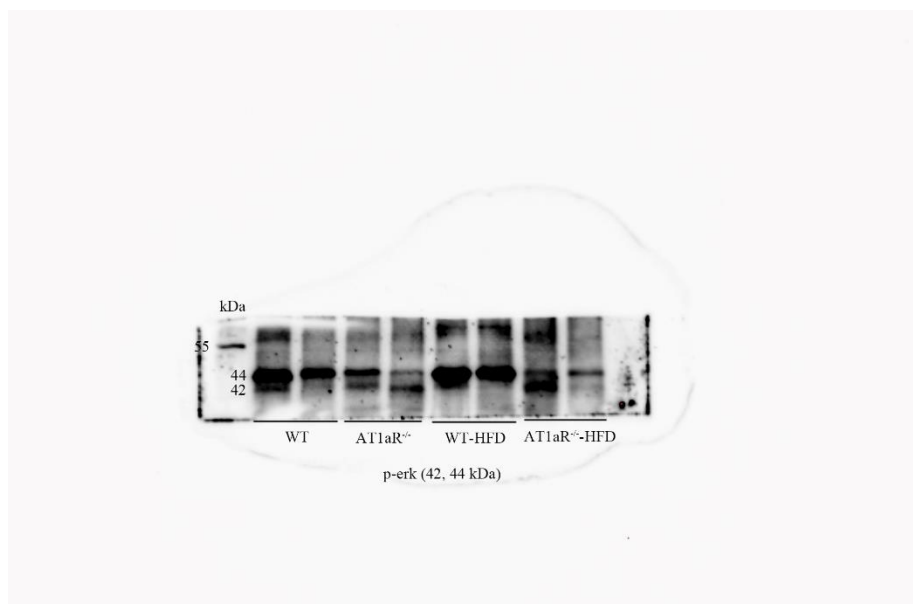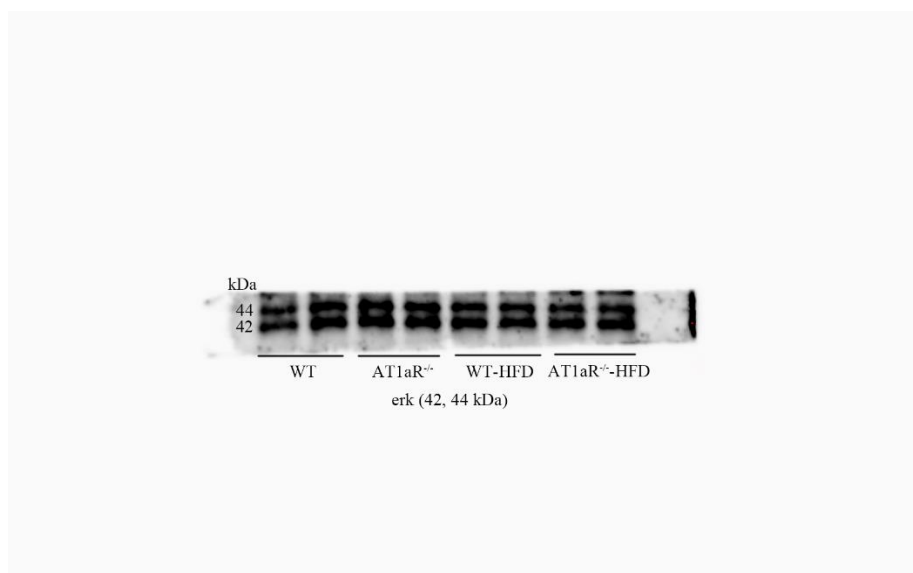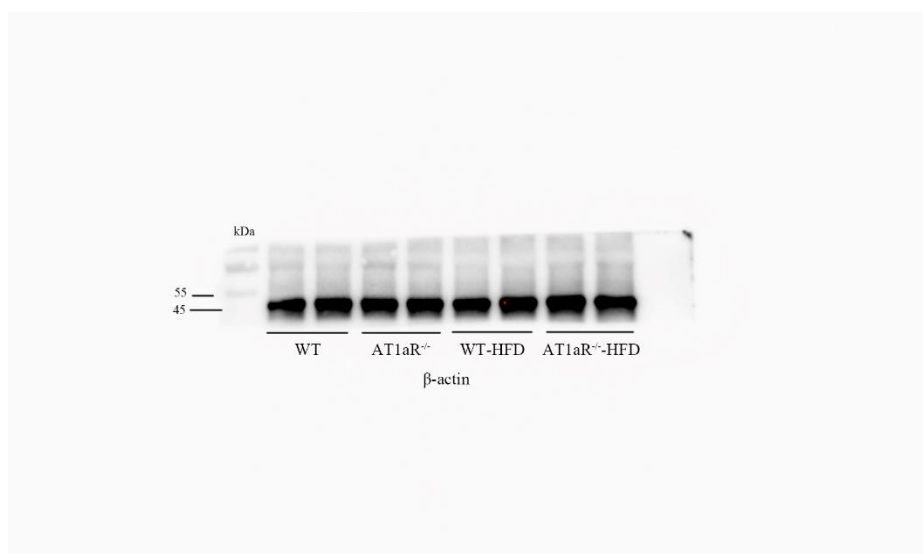

Raw images in Fig 6

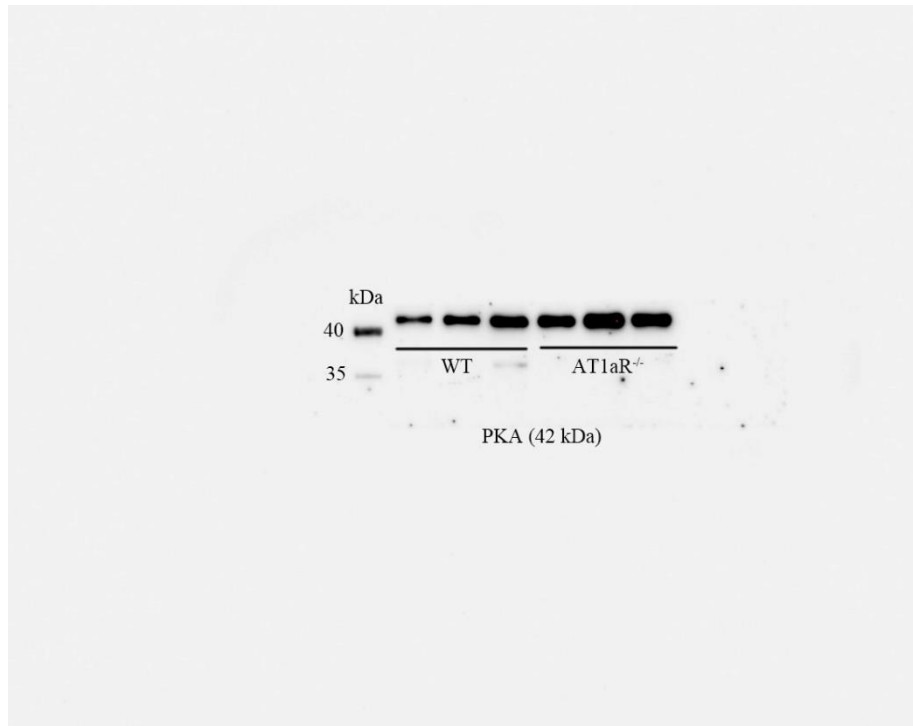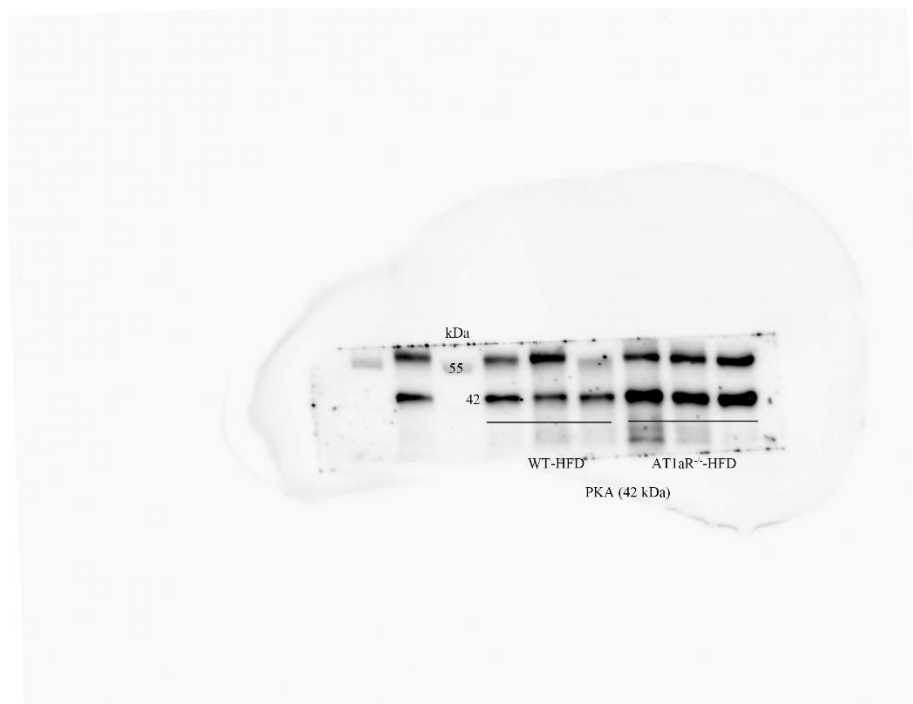

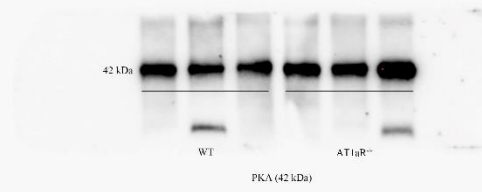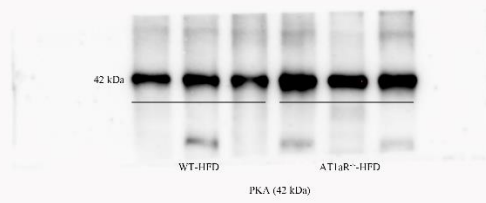

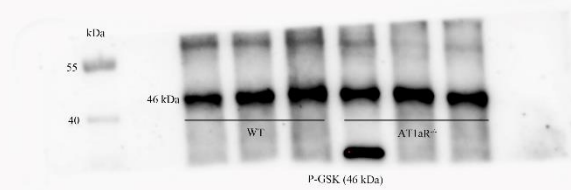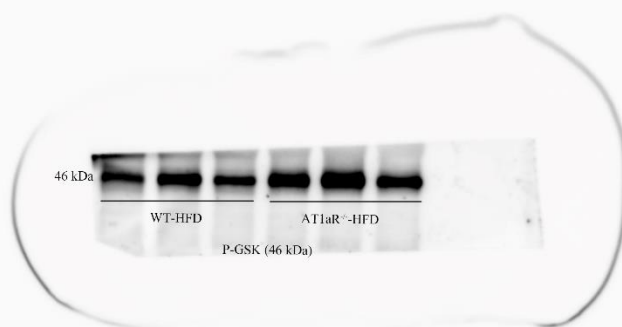

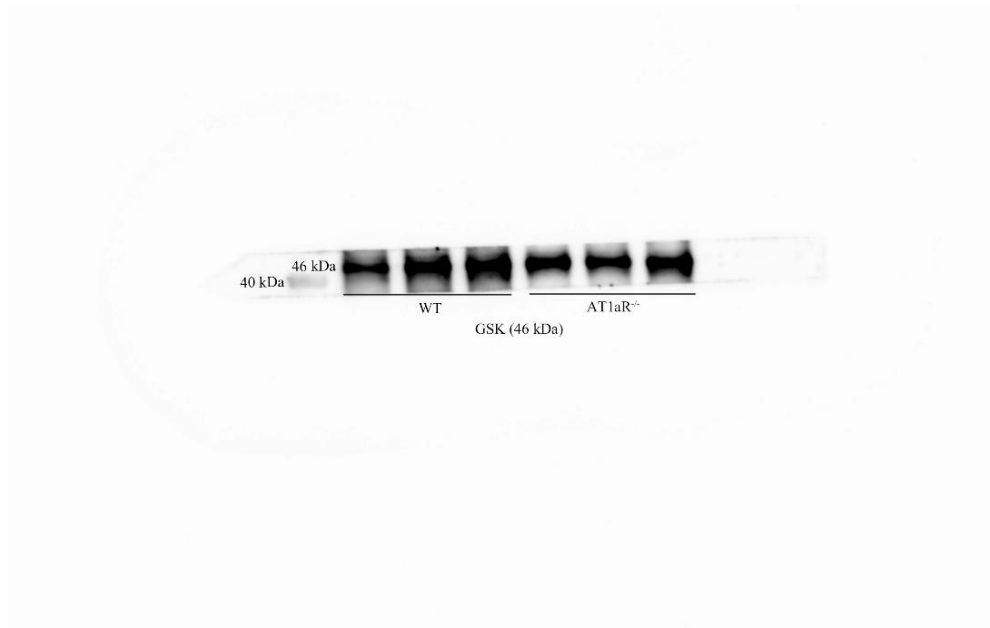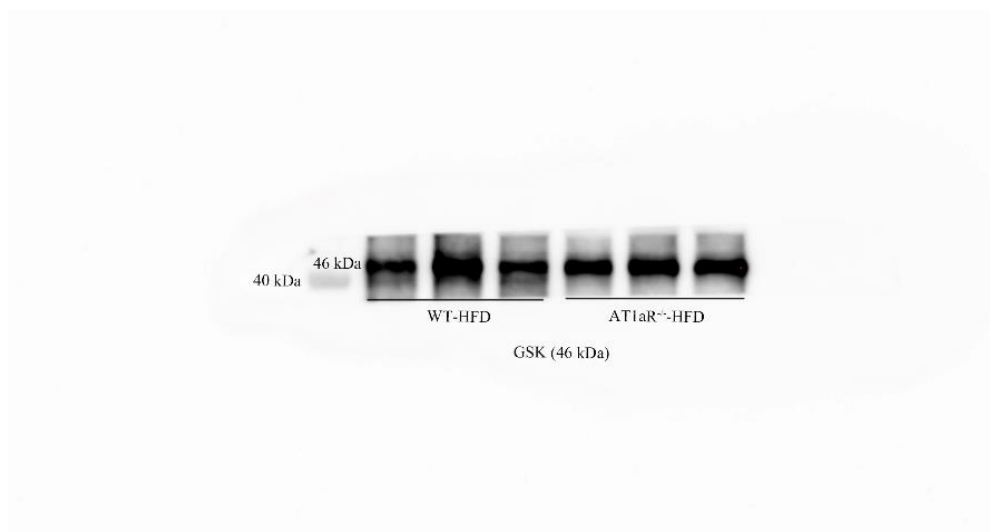

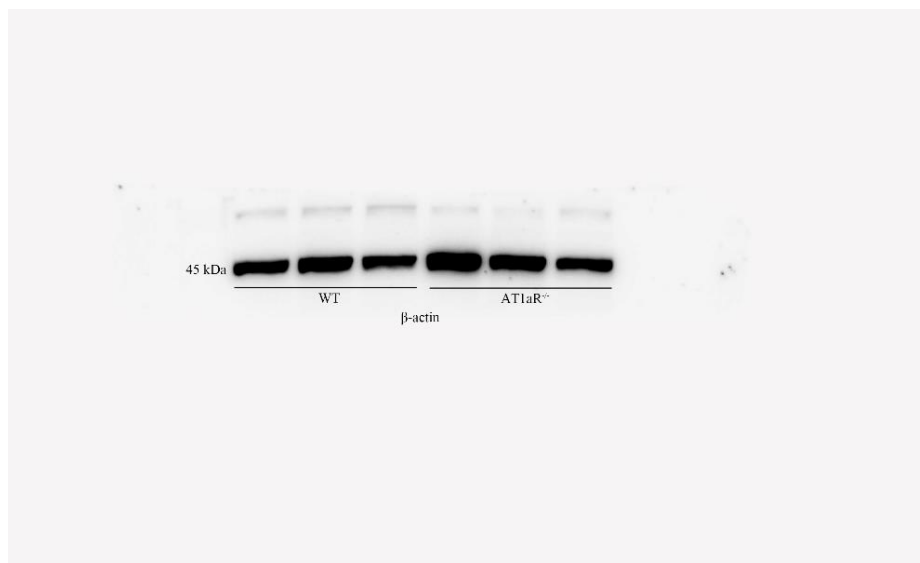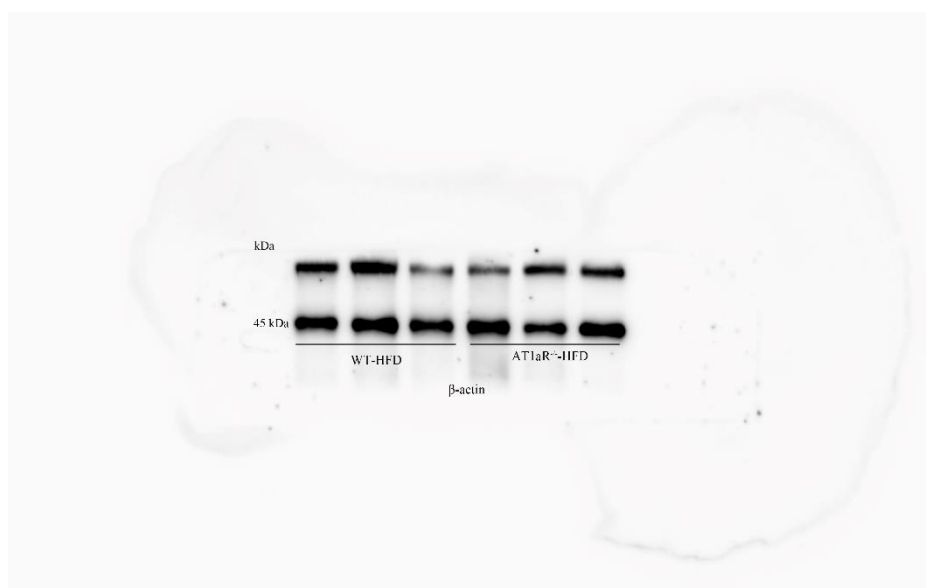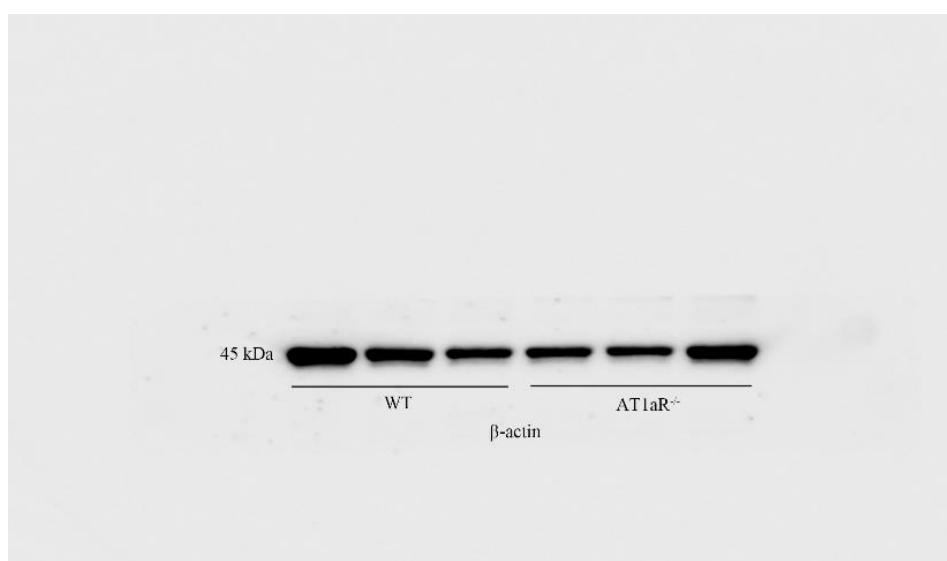

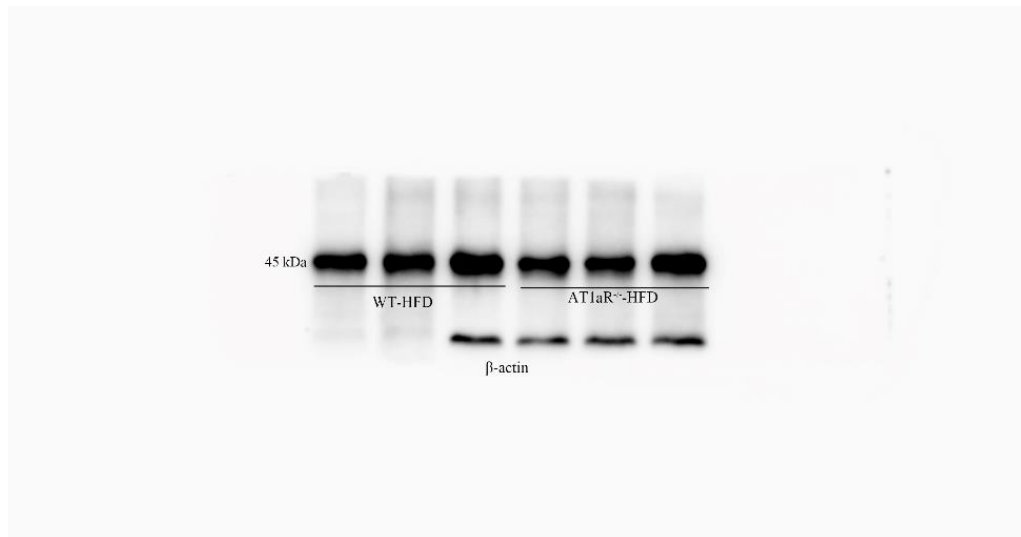

Raw images in Extended Fig 3

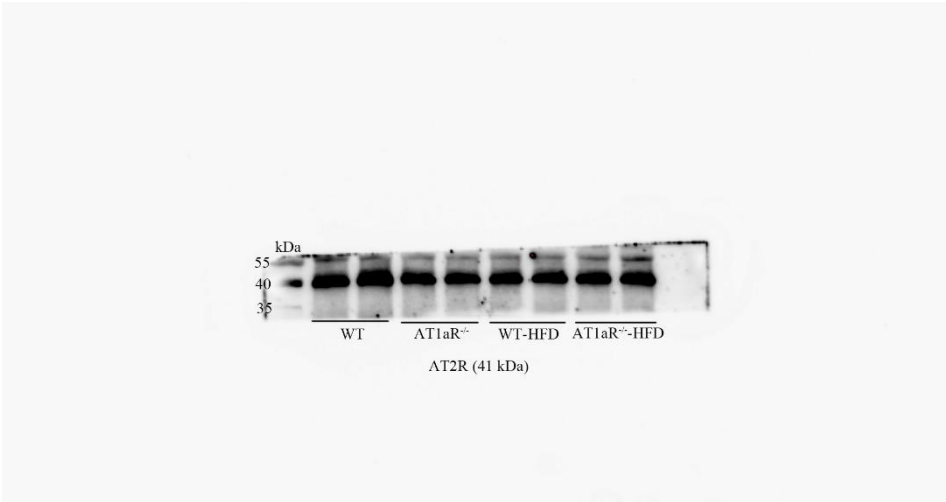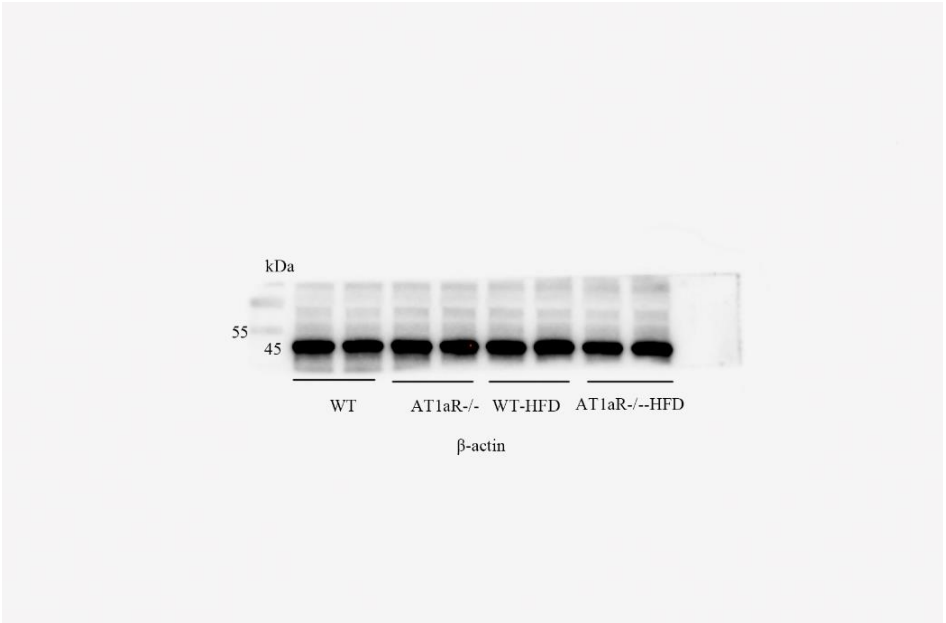

Supplement: S1 Raw images — (PDF) [file pone.0267331.s004.pdf]
